# Data on the degradation of pharmaceuticals and their metabolites by a fungal consortium in a non-sterile stirred fluidized bioreactor

**DOI:** 10.1016/j.dib.2019.105057

**Published:** 2019-12-31

**Authors:** Teddy Kabeya Kasonga, Martie A.A. Coetzee, Ilunga Kamika, Maggy Ndombo Benteke Momba

**Affiliations:** a Department of Environmental, Water and Earth Sciences, Faculty of Sciences, Tshwane University of Technology, P/B X 680, Pretoria, 0001, South Africa; b Department of Environmental Sciences, Faculty of Sciences, University of South Africa, South Africa

**Keywords:** Fungal consortium, Biodegradation, SPE-UPLC/MS, Pharmaceutical compounds

## Abstract

Pharmaceutical compounds (PhCs) are widely prevalent environmental contaminants, with recalcitrant behaviour to conventional biodegradation processes and harmful effects to the ecosystem and human health. Hence, developing an eco-friendly cost-effective process exploring the microbial agents appeared to be promising for the treatment of PhC contaminated effluents. A consortium of the previously isolated and identified South African indigenous fungal strains, namely *Aspergillus niger*, *Mucor circinelloides*, *Trichoderma longibrachiatum*, *Trametes polyzona* and *Rhizopus microspores* was used in a non-sterile stirred fluidized bioreactor (NSFB) to perform the simultaneous biodegradation of selected PhCs. The degradation of the PhCs, namely carbamazepine (CBZ), diclofenac (DCF) and ibuprofen (IBP) as well as their transformation metabolite compounds was carried out using the SPE-UPLC/MS. Here are presented data with regard to the NSFB design, the effect of the concentration of carbon source on the growth of a fungal consortium in the NSFB, the fungal mycelial morphology, and the assessment of the physicochemical parameters. The data displayed the stoichiometric reactions of the transformation fragments and their mass spectrum. For better understanding of the data presented in the present paper, please refer to the original paper “Degradation of pharmaceuticals and their metabolites in non-sterile stirred fluidized bioreactor driven by a fungal consortium” [1].

**Table:** Specifications Table

Subject area	*Microbiology, bioremediation, wastewater management.*
More specific subject area	*Biodegradation of pharmaceuticals in wastewater by a fungal consortium.*
Type of data	*Figures, Graphs.*
How data was acquired	*Zeiss Axio microscope using Zeiss Axiocam ERc 5s camera (Axio Lab, Germany) was used for fungal micrographs. Physicochemical parameters* pH*, dissolved oxygen (DO) and electrical conductivity (EC) were measured using an appropriate electrode of a HACH multi HQ-40d. Ultra-Performance Liquid Chromatography Water Acquity UPLC® system hyphenated to a quadrupole-time-of-flight (QToF) instrument operating with MassLynx™ (version 4.1) software (Waters Inc., Milford, Massachusetts, USA) was used for data acquisition and processing on pharmaceutical degradation and suggestion of metabolite structures.*
Data format	*Raw and Analysed.*
Experimental factor	*The biodegradation of the selected pharmaceutical compounds (PhCs) was conducted in a non-sterile stirred fluidized bioreactor (NSFB) using a consortium of the isolated South African indigenous fungi. The microscopy and the SPE-UPLC/MS were performed to evaluate the fungal micrographs and the PhC removal in the NSFB, respectively.*
Experimental features	*Fungal biomass growth and micrographs, physicochemical parameters and PhC metabolites were evaluated in synthetic wastewater sample from the NSFB. The synthetic wastewater samples contained the PhCs, namely carbamazepine, diclofenac and ibuprofen.*
Data source location	*Tshwane University of Technology and University of Pretoria, Pretoria, South Africa.*
Data accessibility	*All data are presented in this article.*

**Table:** 

**Value of the Data** • The data highlight the design of the non-sterile stirred fluidized bioreactor used for the biodegradation (NSFB) of the selected pharmaceutical compounds (PhCs). • The data provide relevant C/N ratio (carbon source and nitrogen source) needed to growth the fungal consortium in the NSFB. • The data display the monitoring of the physicochemical parameters on the daily basis in the NSFB. • The data demonstrate the UPLC-(+)-QToF-MS spectrum of the metabolites from the selected parent PhCs.

## 1 Data

**Fig. 1:**
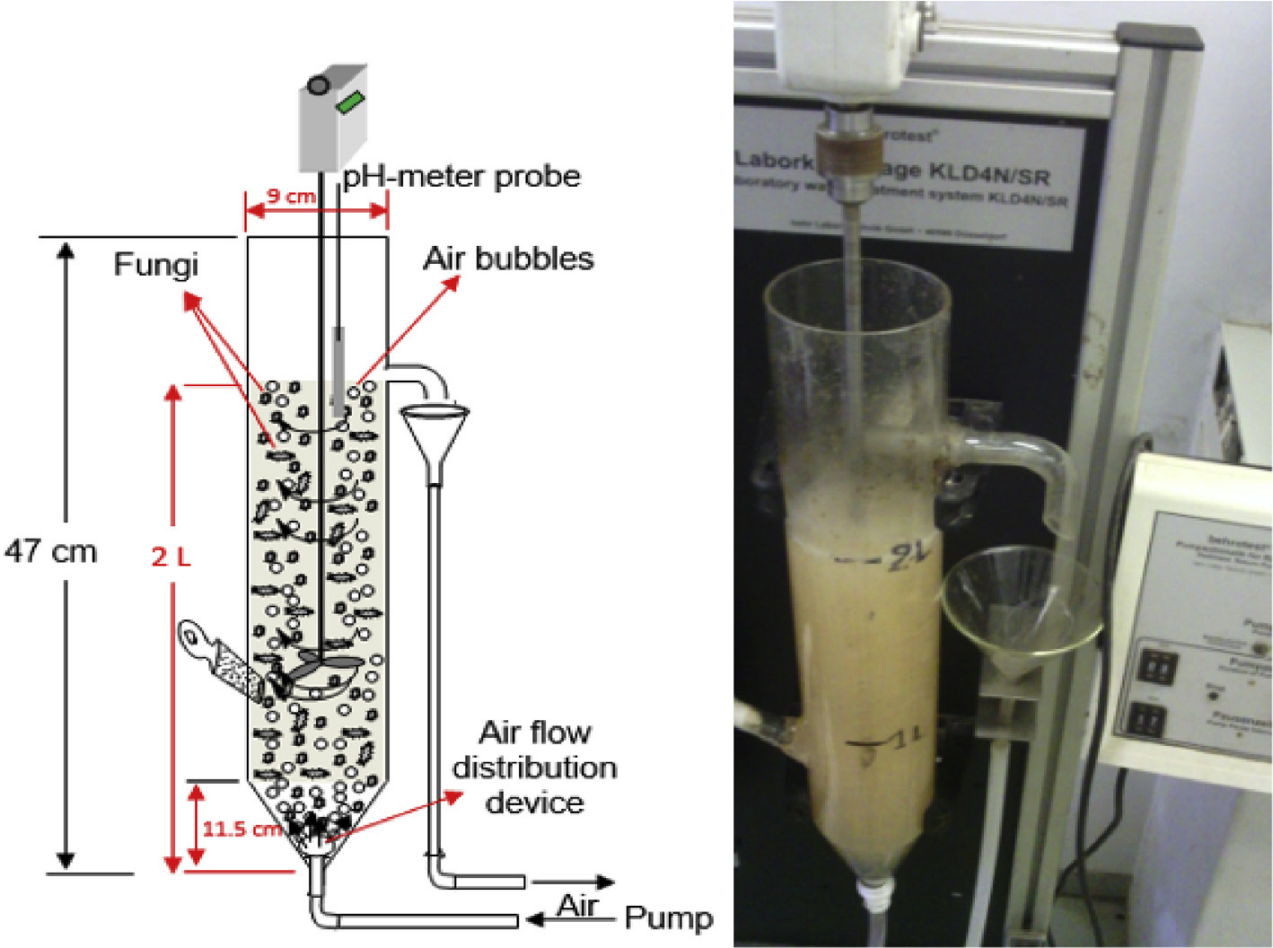
Non-sterile stirred fluidized bioreactor (NSFB) design was used by Kasonga et al. [2] as sequencing batch reactor at a retention time of one and two days.

**Fig. 2:**
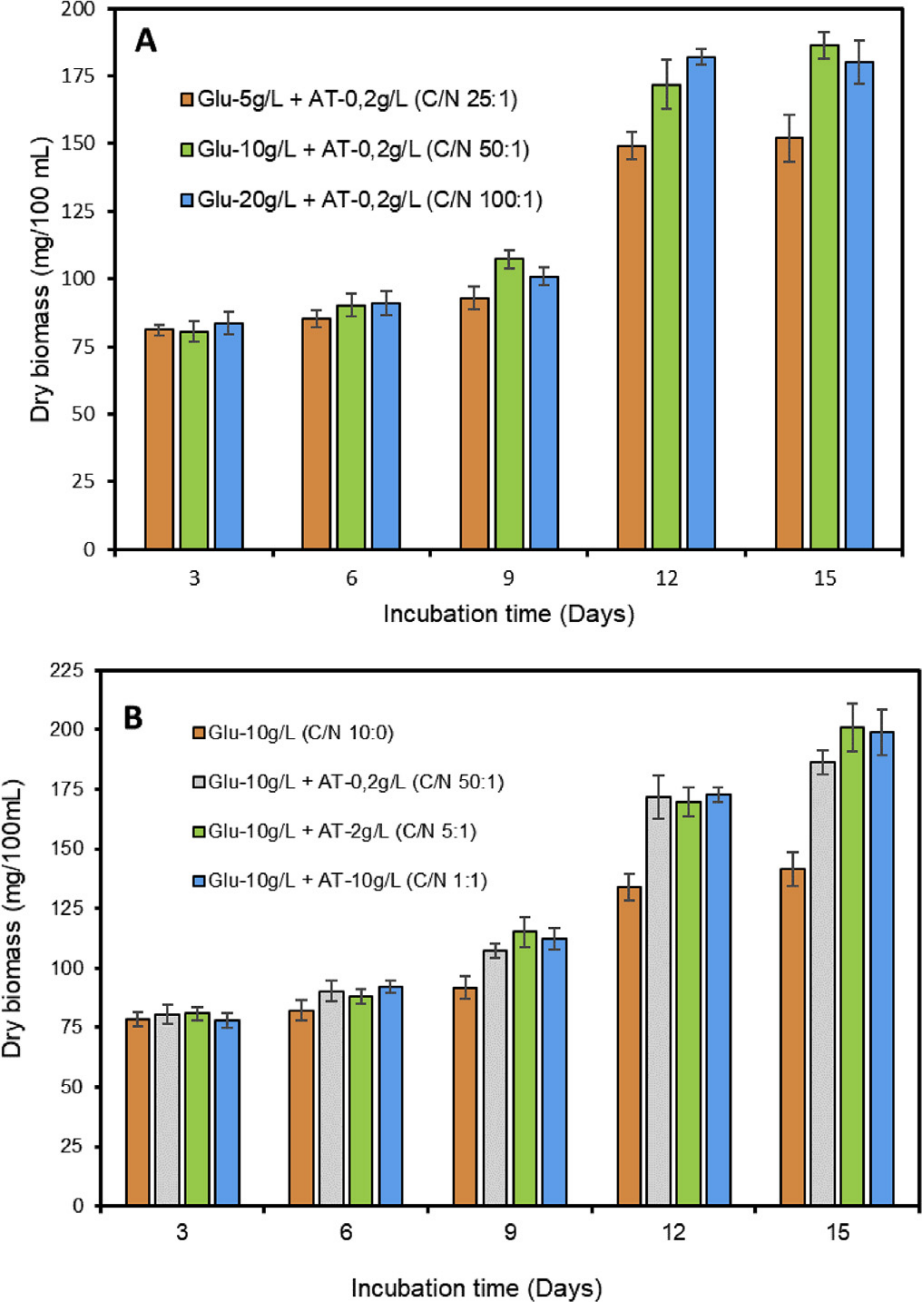
Evaluation of the effect of nutrient source on fungal consortium biomass growth in the NSFB without adjusting the pH of media, with A: carbon source and B: nitrogen source.

**Fig. 3:**
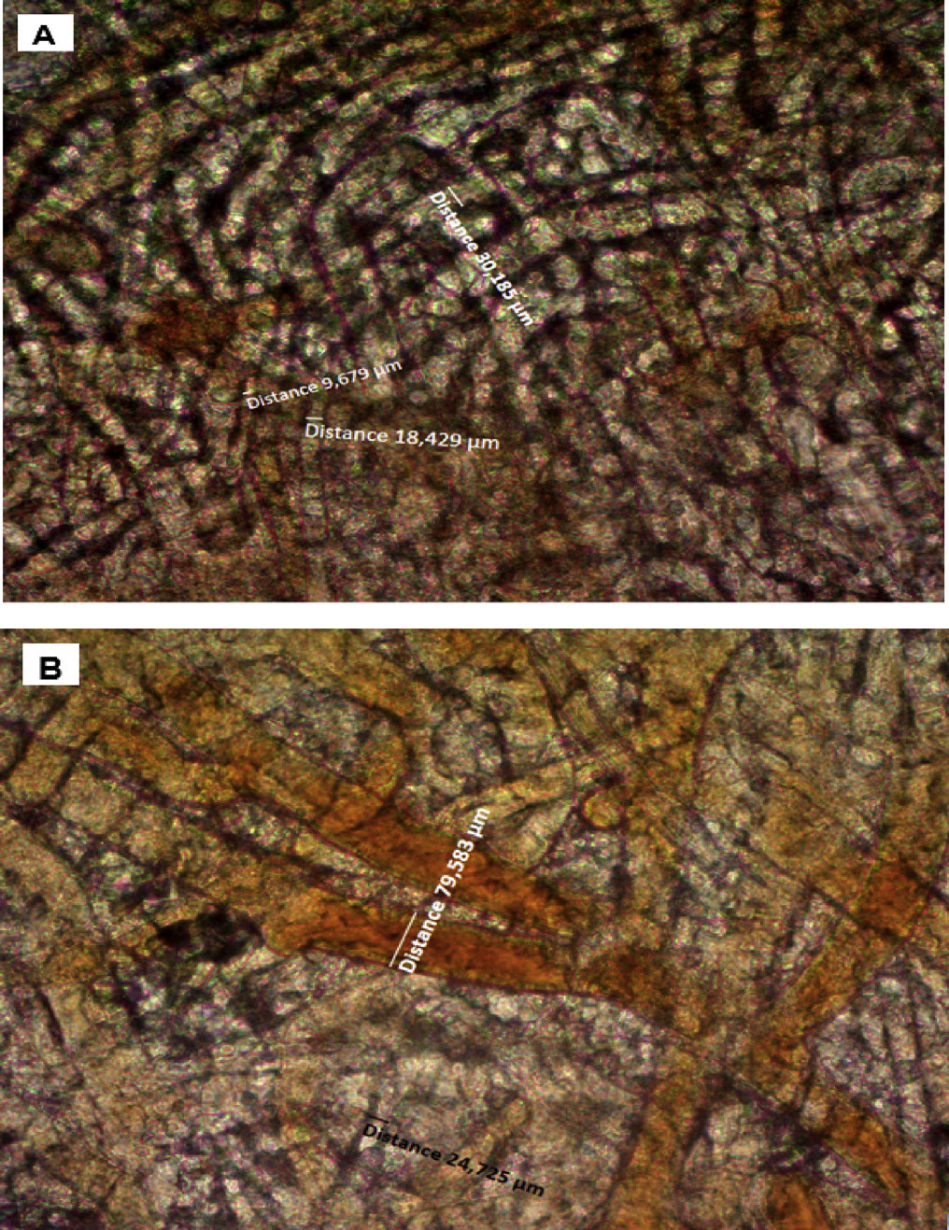
Fungal micrographs indicating the morphological forms of mycelium co-culture in NSFB with Axio Lab objective magnification of A: 10× and B: 40×.

**Fig. 4:**
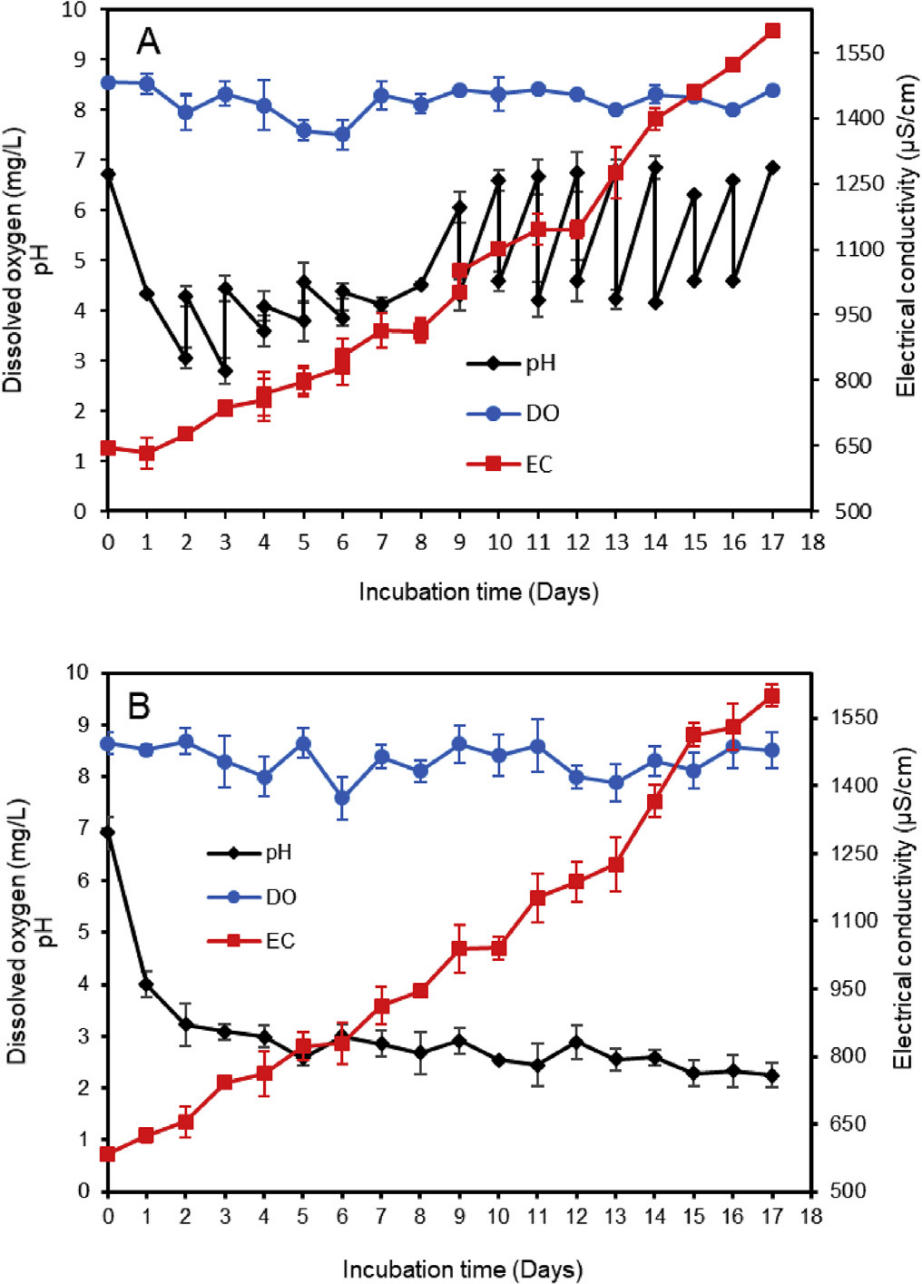
Physicochemical parameters in the NSFB using a consortium of isolated fungal strains while performing the simultaneous removal of selected PhCs, A: with pH adjustment and B: without pH adjustment.

**Fig. 5:**
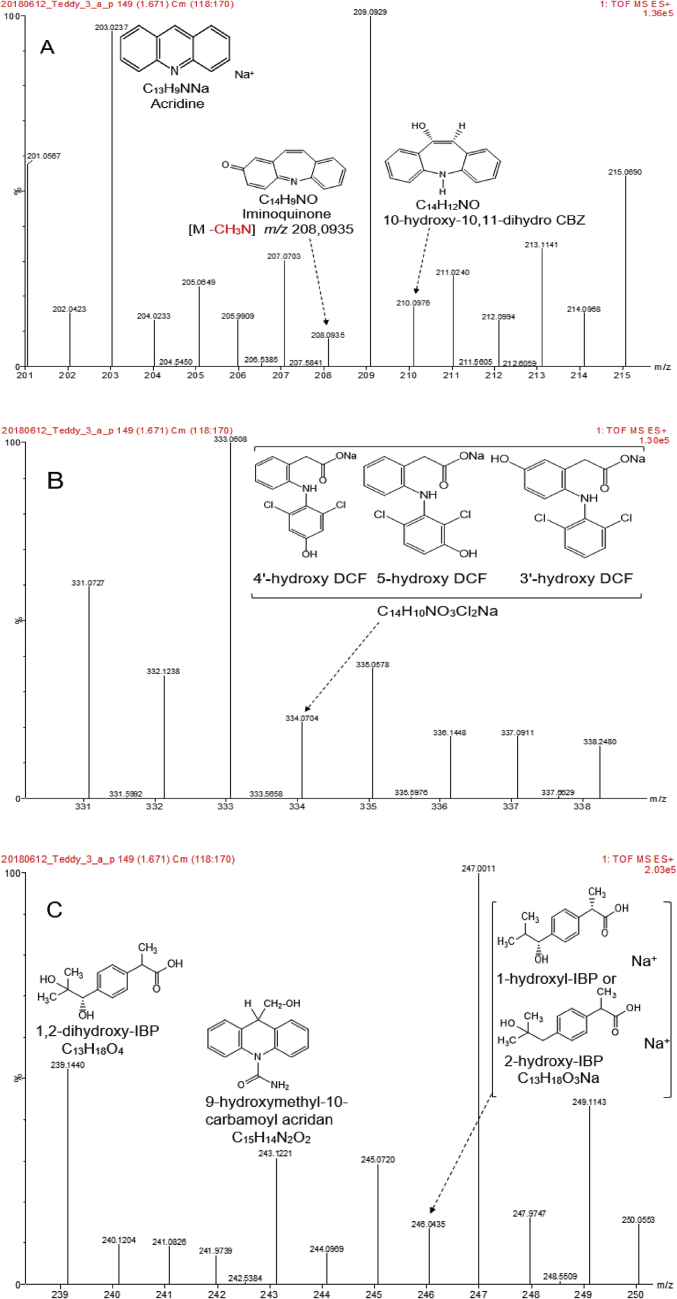
UPLC-(+)-ESI-QToF-MS product ion spectra of the pharmaceutical biodegradation products from wastewater in the NSFB, with A for CBZ, B for DCF and C for IBP.

The occurrence of pharmaceutical compounds (PhCs) and their metabolites with estrogenic activity induced in wastewater effluents have become a big concern. The continuous introduction of these pollutants into the environment is even worsening since PhCs can be transformed into multiple and often unknown by-products, sometimes more toxic than the parent compounds [1]. The biodegradation of these PhCs have attracted scientific over the world. The report in this article provide data on the degradation of PhCs using a consortium of the isolated South African indigenous fungi in a NSFB [1]. Fig. 1 displays a setup of a NSFB used by Kasonga et al. [2]. The NSFB consisted of a 2 L cylindrical glass tube with a conical base was used as a bioreactor (Fig. 1). The 2 L NSFB, with a height of 47 cm, had a diameter of 9 cm and conical size of 11.5 cm enabling the dispersion of air. It was designed to run at room temperature and at a stirrer speed of 120 rpm, under a continuous airlift flow pressure of 8 mmHg provided by an air pump. The air was supplied from the bottom through a multi-pore device ensuring equal distribution of the air. The NSFB containing the synthetic wastewater spiked with the selected PhCs namely CBZ, DCF and IBP was run for a period of 17 day. Fig. 2A and B displays the effect of carbon and nitrogen sources (C/N ratio) on the fungal biomass growth, without pH adjustment. Mycelium morphological form of a consortium of the isolated South African indigenous fungi *Aspergillus niger*, *Mucor circinelloides*, *Trichoderma longibrachiatum*, *Trametes polyzona* and *Rhizopus microspores* is shown in Fig. 3A and Fig. 3B, using the objective magnification of 10× and 40×. Fig. 4A and Fig. 4B provide the evolution of the physicochemical parameters [pH, electrical conductivity (EC) and dissolved oxygen (DO)] in the 2 L NSFB during the removal of PhCs, with and without pH adjustment, respectively. These isolated fungi have exhibited individually the removal of DCF alone in 200 mL batch flasks [3]. A consortium of these fungi was found capable of simultaneous removal of CBZ, DCF and IBP in a sequencing batch reactor [2]. The fungal biodegradation of PhCs has been reported to lead to the metabolite compounds [2,[4], [5], [6]], which might be more toxic than the initial parent compounds [2,7] (see Fig. 5).

## 2 Experimental design, material and methods

### 2.1 Fungal consortium inoculum

The five-isolated indigenous South African fungal mycelium were made individually in low nitrogen medium (LN-m). The composition of LN-m and fungal mycelium preparation were conducted as described Kasonga et al. [2] and by Tien and Kirk [9]. The five indigenous South African fungal strains - *A. niger*, *M. circinelloides*, *T. longibrachiatum*, *T. polyzona* and *R. microsporus* were previously isolated, identified and characterised using the PCR and FEG-SEM methods.

### 2.2 Operation of the non-sterile stirred fluidized bioreactor

The synthetic wastewater in the 2 L NSFB was composed of 10% (/v) or 30% (v/v) of fungal inoculum solution, 10 g/L of D-(+)-glucose anhydrous, 0.2 g/L of ammonium tartrate (AT: Sigma Aldrich, South Africa) at a C/N ratio of 50:1 and 2 g/L of AT for a C/N of 5:1. The selected PhCs, CBZ (Sigma Aldrich, South Africa), DCF (Sigma Aldrich, South Africa) and IBP (Sigma Aldrich, South Africa) were added at a concentration of 1 mg/L of each. A surfactant Tween® 80 (MINEMA Chemicals, South Africa) was added in the NSFB to protect the fungal extracellular enzymes and favour the fungal pellet formation [8]. The consortium inoculum solution consisted of 40 mL or 120 mL of homogenised fungal mycelium of each isolated fungal strains cultured individually as described Tien and Kirk [9] and by Kasonga et al. [2]. The fungal biomass growth was evaluated by weighing its dry biomass per 100 mL as suggested by Patel et al. [10]. The mycelium morphology of the fungal consortium in the NSFB and the physicochemical parameters (pH, EC and DO) were assessed as described in the original paper [1].

### 2.3 Pharmaceutical intermediate compounds

The identification and attribution of the accurate *m/z* of the biotransformation products/intermediates and their sodium adduct generated from parent compounds (CBZ, DCF and IBP) in the NSFB during the biodegradation study were conducted using the SPE-UPLC/MS as described by Kasonga et al. [2,11]. Empirical formula and chemical structures were constructed based on the mass *m/z* of identified intermediates [2] and previously suggested by-product structures of the selected PhCs catalysed by the fungal metabolism [2,4,5].

## Consent to publish

Not applicable.

## Authors’ contributions

TK Kasonga and MAA Coetzee conceived and designed the experiments; TK Kasonga performed the experiments; TK Kasonga and MAA Coetzee analysed the data; MAA Coetzee and MNB Momba contributed reagents/materials/analysis tools; TK Kasonga wrote the paper. MAA Coetzee, I. Kamika and MNB Momba reviewed the paper.

## Ethics approval

This article does not contain any studies concerned with experiment on human or animals.

## Funding

The project “Developing a fungus granulation process for the removal of Endocrine Disrupting Chemicals (EDCs) from wastewater (COE2016/2)” has received financial support from the South African National Research Foundation (NRF) through the grant chair of the Water Quality and Wastewater Management under the SARChI 87310 at the Faculty of Science, Tshwane University of Technology.
